# Domestic tourism demand in the North and the South of Europe in the Covid-19 summer of 2020

**DOI:** 10.1007/s00168-022-01147-5

**Published:** 2022-05-28

**Authors:** Martin Thomas Falk, Eva Hagsten, Xiang Lin

**Affiliations:** 1 School of Business, University of South-Eastern Norway, Bø, Norway; 2 Södertörn University, 141 89, Huddinge, Sweden

**Keywords:** C23, R1, Z3

## Abstract

This study investigates empirically changes in domestic summer tourism demand following the Covid-19 pandemic in 305 regions across six European countries (Denmark, Finland, France, Italy, Spain and Sweden) based on official data. Five different groups of NUTS 3 regions are identified in accordance with a typology suggested by the OECD where density and connectivity are aspects of importance. Dynamic panel data estimations show that large metropolitan regions experience strong decreases in demand (approximately 30 per cent) both in July and August 2020. There are, however, clear differences between the Northern and Southern European countries. In the North, the remote regions encounter an increased demand that is partially offsetting losses in the large metropolitan regions. This pattern cannot be found in the South. The decline in domestic tourism flows to the major metropolitan areas is also more pronounced in the South of Europe, approximately 50 per cent per summer month compared with 20 per cent (July 2020) and stagnation (August 2020) in the North regions.

##  Introduction

During the first pandemic summer of 2020, domestic tourism flows develop unevenly across regions. Rural and mountain regions experience an increase in domestic overnight stays and arrivals, while cities attract fewer visitors (OECD 2020). Several studies examine the progress of domestic tourism flows to different regions during the pandemic (Altuntas and Gok; Arbulú, et al. 2021; Falk et al. 2022), although none of them use a typology that takes into account both connectivity to large metropolitan or urban regions and population density. Since the Covid-19 pandemic affects the perception of crowds (Zenker and Kock 2020) and assemblies are linked to infection rates (Rader et al. 2020), connectivity to and density of regions are aspects of particular importance in analyses of visitor flows.

This empirical study investigates changes in domestic summer tourism demand following the Covid-19 pandemic in 305 regions across six European countries (Denmark, Finland, France, Italy, Spain and Sweden). These countries in the North and in the South of Europe are chosen because of their large domestic markets for summer tourism. They also have long expanses of coastal lines as well as deserted mountainous inland areas (except in Denmark). Five different groups of NUTS 3 regions are identified in accordance with a typology suggested by the OECD where density and connectivity are aspects of importance. This division leads to two metropolitan and three non-metropolitan regions (Fadic et al. 2019). The large metropolitan region includes a city with more than 1.5 million inhabitants (e.g. Barcelona, Bouches-du-Rhône, all departments of Paris, Madrid, Naples, København and surroundings, Rhône, Seville, Stockholm, Turin and Valencia). Domestic summer tourism is approximated by the number of overnight stays based on data from official sources for the months of July and August during the years 2014 to 2020. Dynamic panel data methods are applied to the tourism demand model in which the time effects are allowed to vary across regions.

Many studies explore the determinants of domestic tourism demand based on regional data (Massidda and Etzo 2012; de la Mata and Llano‐Verduras 2012; Patuelli et al. 2013; Massidda and Piras 2015; Campaniello and Richiardi 2018), although most of them use relatively high aggregation levels of data (NUTS 2) and mainly refer to the pre-pandemic period of time. Based on city-level data, Plaza et al. (2015) show that the specific media attention in connection with the opening of the Guggenheim Museum attracts domestic visitors to Bilbao. Population density is a common measure in modelling domestic tourism demand (Massidda and Etzo 2012). This measure characterises rural and urban regions, but does not allow for spatial relationships between regions, for which specific econometric models are required (Romão and Saito 2017).

This study provides a quantification of domestic summer tourism demand across large parts of Europe in times of a worldwide crisis (pandemic) by use of a regional typology where both density and connectivity are built-in aspects of importance (Fadic et al. 2019). Theoretically, the study extends the relationships derived within the fields of medicine and psychology, between crowding, connectivity, population density and infection rates to include also regional tourism flows. The theoretical predictions are contrasted with the observed changes in the tourism flows during the pandemic.

Although the countries differ in size, they have many characteristics in common, making it possible to draw more general conclusions from the study. The Covid-19 pandemic stimulates studies on domestic tourism demand using regional data. This study also contributes to the growing literature in the field by using more detailed NUTS 3 regional data than is commonly employed in the literature (Canale et al. 2019; Altuntas and Gok 2021; Arbulú et al. 2021; Biagi, Brandano and Argiles 2021; Falk et al. 2022).

The implication of the approach used in this study is that not only the opposite ends (urban versus rural) but also the range of regions in between are considered. Another contribution to the literature is the exploration of the possible benefits of using dynamic panel data models explicitly developed for a short time period and a large number of groups as is the case in this study. Dynamic panel data models also have the advantage of controlling for persistence of domestic tourism flows.

After this introductory section, the conceptual background is presented, followed by sections on the empirical approach, data and descriptive statistics, results and concluding remarks.

## Conceptual background

This study focuses on the development of domestic summer tourism demand in the first year of the Covid-19 pandemic (2020). During the summer period, there are fewer pandemic related restrictions on mobility, in particular domestically (Hale 2021).^1^ Literature provides several theories on possible changes in domestic travel flows during the Covid-19 pandemic. Zenker and Kock (2020), for instance, argue that the pandemic influences tourism flows in various ways, such as affecting the image of cities as tourist destinations negatively because they were the first to report high infection rates. Another aspect is that the threat of pathogens makes people more alert and adverse to crowds (Wang and Ackerman 2019; Zenker and Kock 2020). This could cause a change in travel behaviour towards more remote, less populated destinations (Zenker and Kock 2020).

According to Florida et al. (2021), large cities with international airports exhibit much higher infection rates than rural areas during the first phase of the pandemic (see also Nathan and Overman 2020). The higher overall incidence rate and more rapid speed of the virus in densely populated areas are confirmed by the medical literature (Rader et al. 2020). Sy, White and Brooke (2021) show that population density in the USA is significantly positively related to disease transmission, independent of transport connectivity and average income. This suggests that the possibility of effective spread is primarily driven by close contacts in densely populated areas, rather than by connectivity and mobility between different geographical zones. Other studies also demonstrate that urbanisation is the factor most strongly correlated with the number of Covid-19 cases and deaths (Viezzer and Biondi 2021).

In the consecutive phases of the Covid-19 pandemic, the urban–rural divide for infection rates is less pronounced, indicating that the type of density rather than the scale is of importance, compactness of jobs versus residential density, for instance (Florida et al. 2021; Florida and Mellander 2022). Proximity to or connectivity across regions is another possible dimension for the spread of the virus (Florida and Mellander 2022). These aspects are crucial for tourism flows, since it is easier to avoid crowding in remote or disconnected than in urban areas.

Several restrictions on large meetings and events, cancellation of conferences and trade fairs as well as increased use of digital meetings are imposed to curb the spread of the Covid-19 pandemic (Table 1; Hale et al. 2020; 2021; Sharifi and Khavarian-Garmsir 2020).

**Table 1:** Cancellation of public events and restrictions on gatherings. *Source*: Hale et al. 2020; 2021

	Cancellation of public events		Restrictions on gatherings	
	July 2020	August 2020	July 2020	August 2020
Denmark	1	2	3	4
Finland	2	2	1	4
France	1	2	4	4
Italy	2	2	2	4
Spain	2	2	4	4
Sweden	2	2	3	4

Definition of gatherings: 0—no restrictions, 1—restrictions on very large gatherings (> 1000 persons), 2—restrictions on gatherings between 101 to 1000 persons, 3—restrictions on gatherings between 11–100 persons and 4—restrictions on gatherings of 10 persons or less. Definition of public events: 0- no measures, 1—recommend cancelling and, 2—require cancelling

The regulatory measures lead to a decline in business customers, which are particularly common in larger cities and metropolitan regions. Besides the fear of the virus and the need to keep a distance, the partial closure of cultural attractions also makes visiting museums, galleries, restaurants and bars indoors less attractive, even if they are open, something that affects highly populated areas the most.

Early classifications of regions are either based on tourism intensity, continuous measures of population density or on a dichotomy of urban versus rural (Capone and Boix 2008; Santana-Jiménez and Hernández 2011; Marrocu and Paci 2013; Dijkstra and Poelman 2014; Laurin et al. 2020). Veneri and Ruiz (2016) distinguish between predominantly urban, intermediate and primarily rural regions on the basis of population density per local unit. The OECD is involved in several attempts to classify regions that go beyond the simple dichotomy of urban and rural ranging from semi-urbanised to remote peripheral areas (Dijkstra, Poelman and Veneri 2019). Remote peripheral regions, for example, are generally rural in character, but have two distinct characteristics—rurality and geographical remoteness (Brezzi, Dijkstra and Ruiz, 2011). This typology is based on the proximity to functional urban areas and distinguishes between: (i) predominantly urban regions (within a functional urban area), (ii) intermediate regions (close to a functional urban area) or (iii) predominantly rural regions (far from a functional urban area).

In the specific case of analysing domestic demand for summer tourism during a pandemic, a classification is required that takes into account not only urban versus rural, but something more detailed including the degrees of density and possibly also connectivity. Fadic et al. (2019) take the earlier typologies further by identifying five categories of regions including population density, urban accessibility as well as connectivity. Access is defined in terms of the time it takes to reach the nearest urban area, based on both geographical features and the state of the physical road infrastructure.

Thus, the five-region typology allows a detailed examination of domestic tourism flows during different waves of the pandemic (Table 2). This classification is recently used to characterise both the degree of rurality and connectivity and estimations show that only 8 per cent of the OECD population live in remote regions where there is little interaction with nearby cities (Garcilazo, Moreno-Monroy and Martins 2021).

**Table 2:** Typology of regions. *Source*: Fadic et al. (2019)

Region	Description
MRL	Region with a very large city > 1.5 M inhabitants
MRM	Region with a large city > 250 K inhabitants
NMRM	Region near a city > 250 K inhabitants
NMRS	Region near a city of 50 to 250 K inhabitants
NMRR	Remote region where 50 per cent of its population does not have access to any functional urban area within a 60-min drive

The course of events following the Covid-19 pandemic, with mobility and gathering restrictions as well as an initial strong spread of the virus in densely populated areas, as discussed in the literature, is expected to change the pattern of demand for domestic summer tourism in 2020. This change in demand may be apparent, but its extent and variations across regions can only be identified empirically when regional characteristics and other factors of importance are taken into account. Thus, the main hypotheses of the study can be formulated:

H1: Demand for domestic summer tourism exhibits a new pattern in 2020 compared with earlier years.

H2: Demand for domestic summer tourism evolves differently across regions in 2020.

H3: Demand for domestic summer tourism in the North of Europe is affected dissimilarly by the Covid-19 pandemic than in the South.

The validity of the hypotheses is tested on regional data for the three Nordic countries (Denmark, Finland and Sweden) and the three South European countries (France, Italy and Spain) by use of a dynamic panel data model.

##  Empirical model

There are several methodological approaches for analysing changes in regional tourism flows. One popular approach is the shift-share analysis that is commonly used in the field of regional economics (Firgo and Fritz, 2017; Costantino et al. 2021). The advantage of this approach is that it does not require the formulation of an econometric model with its underlying assumptions. A clear disadvantage of shift-share analysis is that different control factors and stochastic factors cannot be taken into account.

An alternative approach presented in the regional economics literature is the tourism gravity model, in which bilateral tourism flows depend on the income of the origin and destination regions, as well as on the actual distance between these two geographical points (De la Mata and Llano-Verduras 2012; Cafiso et al. 2018). Although many merits, this estimation method is seldom applicable for domestic travel flows due to lack of data. Yet another approach derives a regional tourism demand equation depending on prices, real income (GDP) and control factors such as population density (Massidda and Piras 2015). Given the research question and data availability, this latter approach is selected for the present study. Domestic tourism demand depends on economic (real income and prices) as well as non-economic factors.

The literature exhibits several examples where dynamic panel data models are used to estimate the determinants of domestic tourism flows at the regional level (Massidda and Etzo 2012; Canale et al. 2019). These kinds of models have the advantage of controlling for time-invariant regional factors such as natural amenities, which are important determinants of destination attractiveness (Patuelli et al. 2013; Naranpanawa et al. 2019). In dynamic panel data models, it is also possible to control for persistence in tourism demand (Song et al. 2019). By taking the typology of regions into account (Fadic et al. 2019), demand for domestic summer tourism across the six countries is modelled as follows:

$$\begin{aligned} \ln \left( {DNS_{{it}} } \right) & = \theta + \gamma \ln \left( {DNS_{{it - 1}} } \right) + \beta _{1} \ln \left( {Y_{{ct}} } \right) + \beta _{2} \ln \left( {CPI_{{ct}} } \right) + \mathop \sum \limits_{{14}}^{{17}} \lambda _{t} d_{t} + \lambda _{{MRL,20}} d_{{MRL,20}} \\ & + \lambda _{{MRM,20}} d_{{MRM,20}} + \lambda _{{NMRM,20}} d_{{NMRM,20}} + \lambda _{{NMRS,20}} d_{{NMRS,20}} + \lambda _{{NMRR,20}} d_{{NMRR,20}} + \varepsilon _{{it}} , \\ \end{aligned}$$

where ${\text{ln}}\left( {DNS_{it} } \right)$ is the natural logarithm of the number of domestic overnight stays in the region *i* (*i* = 1, 2, …, 305) in the months of July or August of year *t*. $Y_{ct}$ denotes national GDP for the first two quarters in constant prices and $CPI_{ct}$ is the national consumer price index for the first two quarters in country *c*. Parameters $\beta_{1}$ and $\beta_{2}$ are the short-term income and price elasticities of tourism demand. Regional fixed effects are captured by $\alpha_{i} ,$ which is part of the residual $\varepsilon_{it} \left( { \equiv \alpha_{i} + u_{it} } \right)$. A set of time dummy variables $d_{t}$ are introduced to capture presumptive effects of the pandemic in 2020. The years 2018 and 2019 are treated as reference years.

The development in 2020 is modelled as the products of the 5 regional and the 2020 dummies, $d_{MRL,20} , d_{MRM,20} , d_{NMRM,20} , d_{NMRS,20} , {\text{and }}d_{NMRR,20}$ reflecting the variations in kinds of regions in terms of their density, remoteness or connectivity. Parameters of these dummy variables inform if there is a distinct change in demand for domestic summer tourism in 2020, as compared with the average development in the base years 2018–2019.

Due to the correlation between the lagged ln*DNS* and the residual $\varepsilon_{it}$, the ML estimator of the Dynamic Fixed Effects panel data model developed by Hsiao et al. (2002) is employed where all continuous variables are used in their first differences. In comparison with other estimators, such as the GMM, the ML estimator has a better finite sample property (Hayakawa and Pesaran 2015). At the same time, it is designed to fit the datasets with a relatively large number of groups and a short time period, which coincides with the characteristics of the data in this study. This estimator is also robust to the cross-sectional heterogeneity (Hayakawa and Pesaran 2015). A common practice is that the beta coefficients can be interpreted as short-run (semi-) elasticities. Dividing the beta coefficients by 1 minus the coefficient of the lagged dependent variable ($\beta /($ 1−${\upgamma })$) gives the long run elasticity.

An important aspect in any estimation is the presumptive appearance of endogeneity. The possible endogenous relationship between tourism demand, prices, and income is highlighted in the literature (Assaf et al. 2019; Pesämaa et al. 2021). Endogeneity may arise from reverse causality between tourism flows and economic activity. However, by using regional tourism flows and economic activity at the country level, this problem does not appear as tourism flows in a region are unlikely to affect aggregate GDP growth. What is more, the time dummy variables are exogenous, and the OECD classifications referring to population density, remoteness or connectivity remain unchanged at least in the short and medium term.

The specification does not take spatial dependence into account in the estimation method (Patuelli et al. 2013; Romão and Saito 2017; Pompili et al. 2019). This is valid in the period of Covid-19, given the restrictions or advice against travel even to major parts of the neighbouring countries. Another explanation behind this choice is the definition of regions used, that to some extent already includes spatial elements. Results will be presented for two groups of countries, Northern and Southern Europe, as beyond the features in common they also have dissimilarities relating to for instance size, attractiveness, number of regions and climate zone.

## Data and descriptive statistics

This study employs regional panel data on the number of domestic overnight stays in Denmark, Finland, France, Italy, Spain and Sweden for a series of summer months (July and August 2013–2020). Data originate from the national statistical offices in each country and cover 305 regions and 2135 observations. The dependent variable appears in the logarithm of the number of domestic overnight stays for either July or August at the NUTS 3 level. Domestic overnight stays data for Finland, and Sweden are based on all accommodation establishments (hotels, holiday villages, youth hostels, camping sites and commercially arranged private cottages and apartments) while data for Denmark are more limited and only encompass information on hotels with minimum 40 beds.^2^ French data on domestic overnight stays, excluding camping sites, originate from the Statistical Office of France (INSEE).^3^ Spain, as well as Italy, hold registers on hotels and similar accommodation, holiday and other short-stay accommodation, camping grounds, recreational vehicle parks and trailer parks by community and province.^4^

Information on gross domestic product in constant prices refers to the first two quarters of each year, originating from OECDSTAT (Dataset: Quarterly National Accounts: Volume and price indices- GDP expenditure approach, series VOBARSA).^5^ The consumer price information relates to the first six months of each year and is also brought from OECDSTAT.^6^

**Fig. 1:**
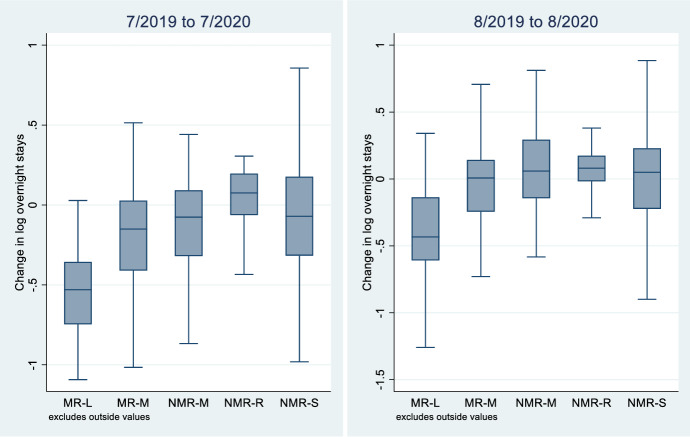
Evolution of domestic overnight stays in 2020. Note: The upper part of the box shows the 75th percentile, the line in the middle the median and the lower line the 25th percentile of the growth rate in domestic overnight stays by region. Source: National statistical offices, see text

Descriptive statistics show that the evolution of domestic overnight stays is uneven across the five regions in the first summer of the Covid-19 pandemic (Table 3A, B, C and Fig. 1). As opposed to the trend of earlier years, large metropolitan regions experience a strong decline in the number of domestic overnight stays in 2020, while remote regions suddenly reverse their development to an increase and thus are the only ones that are growing in July 2020. In August, the downturn is slightly less pronounced, but follows the same pattern as in July, with the exception of the NMRM region in the middle of the scale, which also experiences a slight upswing in demand. There are also apparent variations between the countries in the north and those in the south. Despite the downturn in domestic demand in metropolitan areas, the Northern countries exhibit a slight overall increase in domestic overnight stays both in July and in August compared with the year before. The opposite situation is found for the Southern countries, where demand is contracting in 2020, although the magnitude is somewhat smaller in August. Multivariate tests for equality of the mean values of the five groups show that the change in the number of domestic overnight stays in the summer of 2020 compared with the same period in 2019 differs significantly across regions (Fig. 1,* p*-value < 0.01).

**Table 3:** Average growth of economic variables and domestic overnight stays by region, per cent. *Source*: National statistical offices. OECD Stats

	All regions					
	MR-L	MR-M	NMR-M	NMR-S	NMR-R	All
	July	July	July	July	July	July
*(A)*						
2014	1.7	− 2.6	− 1.0	6.8	− 1.9	− 0.9
2015	5.2	4.9	4.2	0.6	3.0	4.5
2016	0.2	3.8	2.6	5.1	4.3	2.8
2017	6.3	1.6	1.7	1.8	− 0.3	2.5
2018	0.1	− 3.0	0.9	4.5	− 2.1	− 1.6
2019	3.0	− 0.1	− 11.1	− 2.2	− 10.2	− 9.7
2020	− 53.6	− 22.6	− 10.7	− 19.4	6.9	− 12.6
	August	August	August	August	August	August
2014	5.7	− 2.3	− 0.5	13.1	− 1.9	− 0.6
2015	9.0	3.3	2.8	− 0.2	2.6	3.3
2016	− 5.6	2.8	3.6	2.4	3.4	2.1
2017	3.1	0.6	− 0.6	1.8	0.4	1.0
2018	1.2	0.4	1.8	2.1	− 1.2	0.2
2019	12.9	− 0.8	− 9.1	− 0.2	− 3.0	− 4.8
2020	− 41.5	− 4.2	8.6	− 11.1	6.6	− 0.8
	GDPcp	CPI				
2014	0.6	0.5				
2015	1.4	− 0.1				
2016	1.8	− 0.1				
2017	2.1	1.5				
2018	1.8	1.3				
2019	1.6	1.2				
2020	− 11.5	0.4				

	North (Regions in Denmark, Finland, Sweden)					
	MR-L	MR-M	NMR-M	NMR-S	NMR-R	All
	July	July	July	July	July	July
*(B)*						
2014	1.9	1.6	− 0.6	5.1	− 0.7	1.0
2015	8.1	4.2	4.0	− 1.8	− 0.4	1.4
2016	− 1.4	2.9	4.0	4.2	5.4	3.9
2017	2.1	2.4	− 0.2	3.3	− 1.8	0.4
2018	4.7	− 1.3	0.9	− 2.9	− 0.7	− 0.5
2019	8.5	5.3	0.5	2.4	5.5	4.7
2020	− 28.3	− 3.6	4.5	3.3	6.5	0.6
	August	August	August	August	August	August
2014	4.2	1.0	− 1.9	1.2	− 1.3	0.0
2015	9.7	5.7	2.4	3.7	6.5	5.8
2016	0.6	2.0	5.8	2.3	4.1	3.2
2017	− 1.2	2.4	− 3.6	3.8	− 2.2	− 0.4
2018	− 0.8	− 1.1	4.9	− 0.8	− 0.2	− 0.1
2019	18.4	6.0	4.0	− 1.2	3.9	4.8
2020	− 17.2	4.9	13.6	− 1.5	2.2	1.2
	GDPcp	CPI				
2014	1.1	0.6				
2015	2.3	0.3				
2016	2.6	0.5				
2017	2.9	1.2				
2018	2.1	1.2				
2019	1.8	1.4				
2020	− 3.8	0.5				

	South (Regions in France, Italy and Spain)					
	MR-L	MR-M	NMR-M	NMR-S	NMR-R	All
	July	July	July	July	July	July
(C)						
2014	1.6	− 3.2	− 1.0	0.1	− 2.7	− 1.2
2015	4.5	4.9	4.2	5.9	5.0	5.1
2016	0.6	4.0	2.4	1.6	3.7	2.6
2017	7.4	1.5	1.9	4.6	0.8	3.0
2018	− 1.0	− 3.2	1.0	− 2.1	− 3.1	− 1.8
2019	1.6	− 0.9	− 12.1	− 21.7	− 21.0	− 12.6
2020	− 59.9	− 25.3	− 12.1	− 7.6	7.2	− 15.2
	August	August	August	August	August	August
2014	6.1	− 2.8	− 0.4	− 0.3	− 2.3	− 0.7
2015	8.8	2.9	2.9	2.2	0.2	2.8
2016	− 7.2	3.0	3.4	1.7	3.0	1.9
2017	4.2	0.4	− 0.3	1.9	2.3	1.3
2018	1.7	0.6	1.5	− 0.2	− 1.9	0.3
2019	11.6	− 1.8	− 10.3	− 12.1	− 7.8	− 6.7
2020	− 47.6	− 5.5	8.2	2.8	9.7	− 1.2
	GDPcp	CPI				
2014	0.6	0.5				
2015	1.2	− 0.2				
2016	1.6	− 0.2				
2017	2.0	1.6				
2018	1.7	1.4				
2019	1.5	1.1				
2020	− 13.0	0.3				

GDP relates to constant prices in the national currency.

## Empirical results

Dynamic panel data estimations for the 305 regions reveal that there is a change in the pattern of domestic summer tourism demand in the pandemic summer of 2020 (Tables 4 and 5). The long-term increase in demand for summer tourism to large metropolitan areas (MRL) is broken both in the South and the North of Europe, while tourism flows to remote regions (NMRS and NMRR) increase. Given the high degree of persistence, the interpretation focuses on the short-term effects. Coefficients for metropolitan areas are approximately -0.34, suggesting that the number of domestic overnight stays is 30 per cent lower in the short term than in the reference period. Estimates of the dummy variables for the remotest regions (NMRR) are 0.20 in July and 0.12 in August. This implies that domestic tourism demand increases in the short-run by 18 and 12 per cent, respectively (calculated according to Halvorsen and Palmquist 1980).

**Table 4:** Demand for domestic overnight stays in July, Dynamic panel data estimations*. * *Source*: National statistical offices, OECD Stats and own calculations

	All regions	South	North
ln(*dns*)(*t*-1)	0.492***	0.504***	0.564***
	(0.09)	(0.10)	(0.10)
ln(*gdp*)	1.277***	0.244	2.135***
	(0.26)	(0.42)	(0.61)
ln(*cpi*)	− 2.176***	− 1.889*	− 1.720***
	(0.61)	(1.03)	(0.44)
NMRR 2020	0.201***	0.097	0.159***
	(0.04)	(0.08)	(0.03)
NMRS 2020	0.068	− 0.059	0.119***
	(0.05)	(0.07)	(0.04)
NMRM 2020	0.064	− 0.065	0.095***
	(0.04)	(0.07)	(0.04)
MRM 2020	− 0.032	− 0.163**	0.039
	(0.05)	(0.08)	(0.05)
MRL 2020	− 0.335***	− 0.493***	− 0.217**
	(0.07)	(0.09)	(0.09)
Year dummy 2017 (reference 2018 + 2019)	0.064***	0.061***	0.013
	(0.01)	(0.01)	(0.02)
Year dummy 2016	0.046**	0.019	0.071**
	(0.02)	(0.03)	(0.03)
Year dummy 2015	0.066***	0.027	0.087**
	(0.02)	(0.03)	(0.04)
Year dummy 2014	0.037*	− 0.023	0.122**
	(0.02)	(0.03)	(0.05)
Constant	− 2.271	11.029**	− 16.840**
	(3.21)	(4.78)	(6.84)
Number of observations	2135	1778	357
Number of regions	305	254	51

Asterisks ***, ** and * denote significance at the 1, 5 and 10 per cent levels. The standard errors are reported within the parentheses. The estimations are based on robust standard errors and conducted using the Stata command xtdpdqml (Kripfganz, 2016) that implements the QML method of Hsiao et al. (2002). The regions are defined as the following: with a very large city > 1.5 M inhabitants (MRL), with a large city > 250 K inhabitants (MRM), near a city > 250 K inhabitants (NMRM), near a city between 50 and 250 K inhabitants (NMRS) and remote region (NMRR). South means regions in France, Italy and Spain while North implies regions in Denmark, Finland and Sweden

**Table 5:** Demand for domestic overnight stays in August, Dynamic panel data estimations. *Source*: National statistical offices, OECD Stats and own calculations

	All countries	South	North
ln(*dns*)(*t*-1)	0.623***	0.616***	0.909***
	(0.12)	(0.12)	(0.09)
ln(*gdp*)	0.249	− 0.658	3.802***
	(0.60)	(0.92)	(0.58)
ln(*cpi*)	− 1.448**	− 0.376	− 3.680***
	(0.60)	(0.92)	(0.58)
NMRR 2020	0.115***	0.028	0.181***
	(0.03)	(0.06)	(0.03)
NMRS 2020	0.055	− 0.057	0.153***
	(0.05)	(0.07)	(0.05)
NMRM 2020	0.123**	0.001	0.229***
	(0.05)	(0.07)	(0.09)
MRM 2020	− 0.016	− 0.109	0.168**
	(0.05)	(0.07)	(0.08)
MRL 2020	− 0.341***	− 0.511***	− 0.119
	(0.09)	(0.11)	(0.11)
Year dummy 2017 (ref 2018 + 2019)	0.007	0.016	0.015
	(0.01)	(0.01)	(0.02)
Year dummy 2016	− 0.007	− 0.006	0.112**
	(0.02)	(0.03)	(0.04)
Year dummy 2015	− 0.001	− 0.016	0.212***
	(0.02)	(0.03)	(0.04)
Year dummy 2014	0.033	− 0.056**	0.231***
	(0.02)	(0.03)	(0.07)
Constant	7.623**	15.789***	− 35.342***
	(3.33)	(4.84)	(8.04)
Number of observations	2135	1778	357
Number of regions	305	254	51

Asterisks ***, **, and * indicate the significance at 1%, 5%, and 10%, respectively. Points estimates are reported for coefficients. The standard errors are reported in the parentheses. The significance is based on the t-test. The regions are defined as the following: with a very large city > 1.5 M inhabitants (MRL), with a large city > 250 K inhabitants (MRM), near a city > 250 K inhabitants (NMRM), near a city between 50 and 250 K inhabitants (NMRS) and remote region (NMRR). See Table 4

Overall, the results indicate that not only population density but also the degree of connectivity to urban areas influences domestic tourism demand when a sudden shock like the Covid-19 pandemic occurs. This is a new finding in the literature. Thus, the first two hypotheses cannot be rejected.

Hidden behind the overall figures, there are more pronounced variations between regions in the South and the North of Europe. This means that the third hypothesis cannot be rejected either. In July, both metropolitan regions (MRL and MRM) in the South experience a decrease in demand for tourism, although the impact is strongest on the larger regions. As opposed to the regions in the North, there is also no direct significantly positive effect on the overnight stays in the three remote regions in the South. In the North, these three remote regions are all gaining in overnight stays, while the next largest metropolitan area is not directly affected and only the largest regions experience a decline.

The decrease in demand for the largest metropolitan regions in the South is equally strong in August, while there is no longer a significant direct effect in the North, as compared with the reference period. Instead, the demand for overnight stays surges in all regions in the group of Northern countries, but the effect is no longer strongest in the utmost remotest areas, but in the third remotest regions (NMRM).

Coefficients for real GDP and CPI show the expected signs, but the significances are weaker for the Southern countries. However, the income and price elasticities should be interpreted with caution as they only vary across time and countries. The coefficients for lagged domestic overnight stays range between 0.50 and 0.91 indicating a relatively high degree of persistence, which is also consistent with previous studies (Gil-Alana and Huijbens 2018; Falk et al. 2022). Besides this, there are no known similar studies to compare present results with.

As a robustness check, the same specification is estimated with four classifications (three non-metropolitan and one metropolitan region). Unreported results show that these estimations are largely consistent with those presented. In addition, other dynamic panel data methods are used such as the system GMM estimator (Blundell and Bond 1998; Roodman 2009). These estimations give similar results and are available upon request. However, results for the Nordic sample should be interpreted with caution as the system GMM estimator requires a relatively large number of groups which is not fully satisfied for this area.

## Conclusion and discussion

This study investigates changes in domestic summer tourism demand following the Covid-19 pandemic in 305 regions across six European countries (Denmark, Finland, France, Italy, Spain and Sweden) based on official data. These countries are chosen because of their large domestic markets for summer tourism. They also have long expanses of coastal lines as well as deserted mountainous inland areas (except Denmark). Literature in medicine and psychology on the relationship between crowding, connectivity, population density and infection rates is used to formulate the hypotheses on the shift in regional tourism flows. Five different groups of NUTS 3 regions are identified in accordance with a typology suggested by the OECD where density and connectivity are aspects of importance. Dynamic panel data estimations reveal that the pattern of domestic tourism demand during the summer of 2020 is uneven across regions and differs from the trend of earlier years. Large metropolitan regions face a marked decline of more than 30 per cent in both July and August, when real GDP (capturing the impact of the Covid-19 recession), prices and the level of overnight stays in the same summer months earlier years are controlled for. In contrast, remote areas experience a short-term increase of 18 per cent in July and 12 per cent in August.

The downturn in domestic tourism demand for metropolitan areas can partly be explained by the absence of events and attractions due to restrictions with the purpose to calm down the pandemic. Densely populated areas could also become frightening for visitors per se. In such situations, rural and remote regions offer more space and opportunities for individual outdoor activities, which might be far more appealing. However, only in the North, there are indications of a substitution in demand from metropolitan to remote areas. While all countries experience a pronounced downturn in demand for tourism to large metropolitan areas, only the countries in the North meet increased demand for remote areas. The magnitude of the decline is also larger for the regions in the South of Europe.

Several conclusions can be drawn from the study. First, regional typology indicators are stronger determinants of domestic tourism and travel demand during the Covid-19 pandemic than classical economic factors such as income and prices. Second, an in-depth distinction of different types of regions that integrate more aspects than population density is important. Third, the results suggest that more aggregated regional data such as the NUTS 2 level are not sufficient to explain variations in domestic travel flows.

Some limitations need to be mentioned. The study covers a short time period, only the first summer of the Covid-19 pandemic. Domestic tourism demand may vary over different stages of the pandemic. Future work should apply the model to other regions of the world. In addition, the remote region can be further refined by distinguishing between coastal and mountain regions.

## Footnotes

1 Covid-19 Oxford stringency index, category public events, Source: https://data.humdata.org/dataset/oxford-covid-19-government-response-tracker

2 SE: https://www.statistikdatabasen.scb.se; /pxweb/en/ssd/START__NV__NV1701__NV1701B/NV1701T910M/. DK: https://www.statbank.dk/statbank5a/default.asp?w=1280; FI: http://pxnet2.stat.fi/PXWeb/pxweb/en/StatFin/StatFin__lii__matk/statfin_matk_pxt_11iz.px/;

3 (https://www.insee.fr/en/statistiques?debut=0&theme=67).

4 https://www.ine.es/jaxiT3/Tabla.htm?t=2941 and http://dati.istat.it/?lang=en&SubSessionId=2f40698c-c301-4e60-9a25-99fd73adf5bb.

5 https://stats.oecd.org/index.aspx?queryid=60702#.

6 https://stats.oecd.org/viewhtml.aspx?datasetcode=PRICES_CPI&lang=en.
